# Supplementary material for: L1CAM is required for early dissemination of fallopian tube carcinoma precursors to the ovary
Source: Commun Biol. 2022 Dec 12;5:1362. doi: 10.1038/s42003-022-04314-8 (PMC9744873; doi:10.1038/s42003-022-04314-8)
Supplement: Supplementary file 2 — Supplementary Information [file 42003_2022_4314_MOESM2_ESM.pdf]

L1CAM is required for early dissemination of fallopian tube carcinoma precursors to the ovary

Kai Doberstein[1,#], Rebecca Spivak[1], Hunter D. Reavis[1], Jagmohan Hooda[1,^], Yi Feng[1], Paul T. Kroeger Jr[1], Sarah Stuckelberger[1], Gordon B. Mills[2], Kyle M. Devins[3], Lauren E. Schwartz[3], Marcin P. Iwanicki[4], Mina Fogel[5], Peter Altevogt[6], Ronny Drapkin[1,7,*]

Affiliations

[1] Ovarian Cancer Research Center, University of Pennsylvania, Perelman School of Medicine, Philadelphia, PA USA 19104

[2] Knight Cancer Institute, Oregon Health and Science University, Portland, OR USA 97239

[3] Department of Pathology and Laboratory Medicine, University of Pennsylvania, Perelman School of Medicine, Philadelphia, PA USA 19104

[4] Department of Bioengineering, Chemistry, Chemical Biology and Biological Sciences, Stevens Institute of Technology, Hoboken, NJ USA

[5] Central Laboratories, Kaplan Medical Center, Rehovot, Israel

[6] Skin Cancer Unit, German Cancer Research Center (DKFZ), Heidelberg, Germany

[7] Basser Center for BRCA, Abramson Cancer Center, University of Pennsylvania, Perelman School of Medicine, Philadelphia, PA USA 19104

[#] Present address: Department of Gynecology, Medical Faculty Mannheim of the Heidelberg University, Mannheim, Germany

^ Present address: University of Pittsburgh, Hillman Cancer Center, Pittsburgh, PA USA

* Corresponding author:

Ronny Drapkin, Ovarian Cancer Research Center, Perelman School of Medicine, University of Pennsylvania, Philadelphia, PA 19104, USA

Email: rdrapkin@pennmedicine.upenn.edu; phone: +1-215-746-3973

Supplementary Figure 1

a

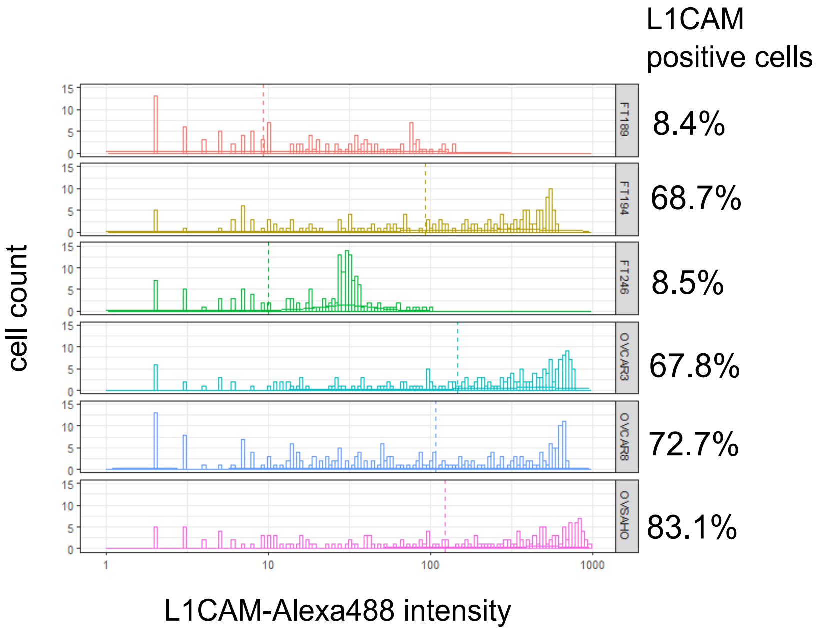

b

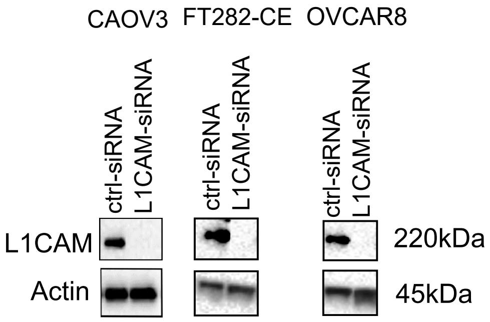

c

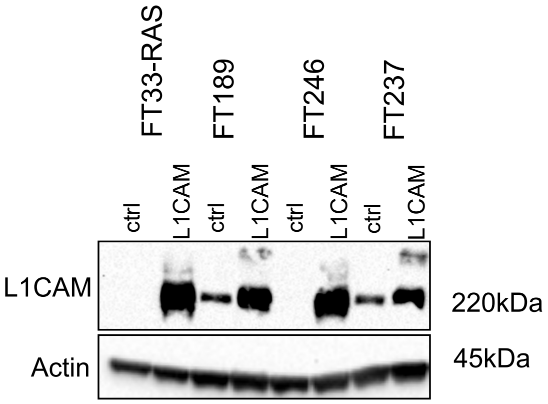

Supplementary Figure 1:

(a) Histogram of flow cytometry analysis of surface bound L1CAM measured with the L1CAM 11A antibody depicted as cell counts relative to L1CAM fluorescence intensity. Numbers on the y-axis depict the percentage of L1CAM positive cells relative to background control for each cell line. (b) Western blot analysis with antibodies against L1CAM and beta-Actin of CAOV3, FT282-CE and OVCAR8 after siRNA knock-down of L1CAM. (c) Western blot analysis with antibodies against L1CAM and beta-Actin of FT33-RAS, FT189, FT246 and FT237 after transfection with a control vector or a vector expressing L1CAM.

Supplementary Figure 2

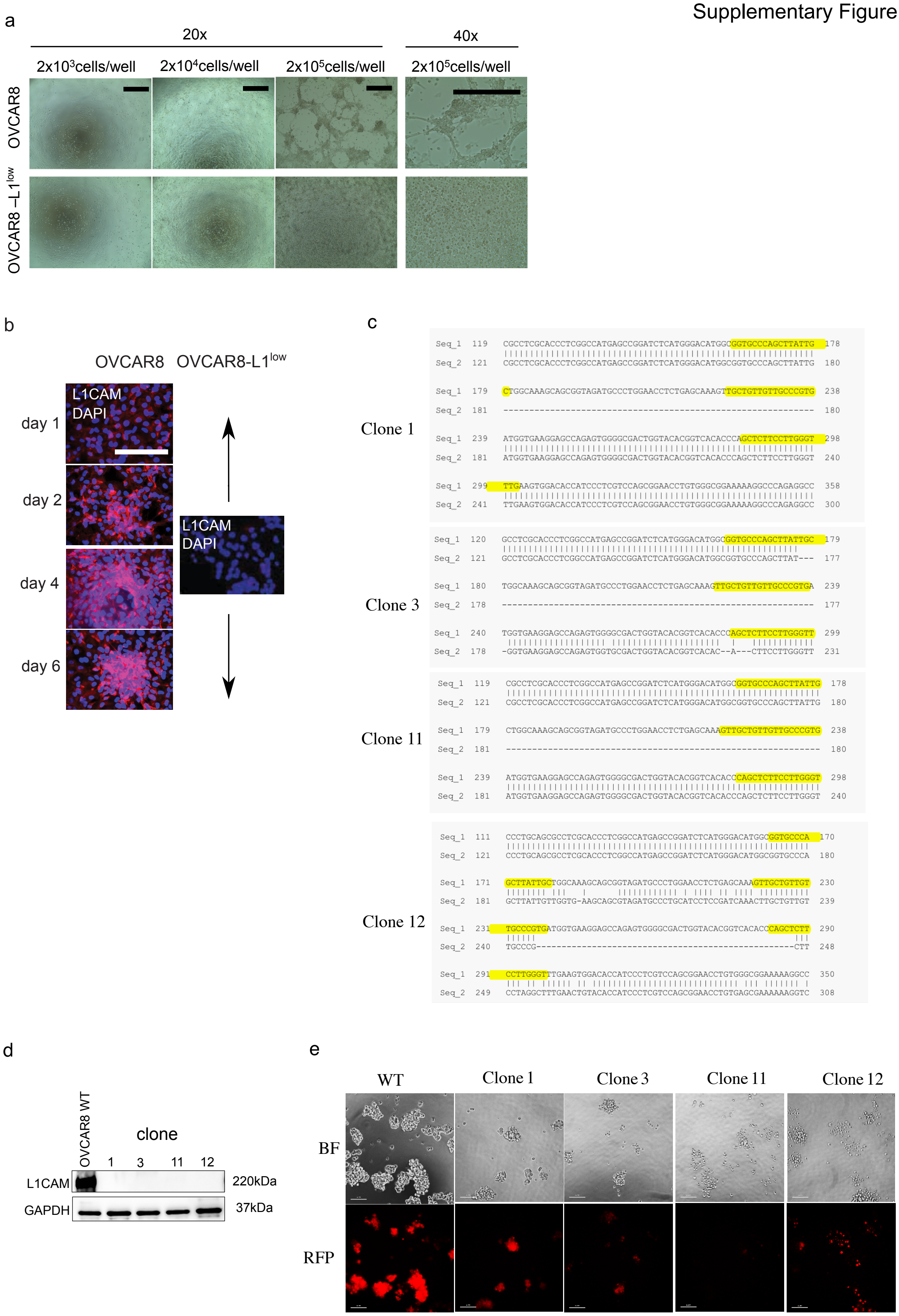

Supplementary Figure 2:

(a) Bright field images of OVCAR8 (upper panel) and OVCAR8-L1^{low} (lower panel) cells seeded at increasing densities for 72 h in 24 well plates. Scalebar represents 200 µm for 20x and 10x objective and 1mm for 4x objective. (b) Fluorescent images of OVCAR8 and OVCAR8-L1^{low} representing L1CAM (red) and DNA (DAPI, blue) at different time points. Scalebar represents 100 µm. (c) Genomic DNA of knockout clones (1, 3, 11, 12) was sequenced near the site of CRISPR/Cas9-induced cleavage in the L1CAM locus to confirm genome editing. Seq1 represents the wild type OVCAR8 and Seq2 the sequence of the representative clone. The guide RNA target sequences are marked yellow. (d) Western blot analysis with antibodies against L1CAM and beta-Actin of OVCAR8 wild type and L1CAM knockout clones 1, 3, 11 and 12. (e) upper lane: bright field (BF) and lower lane: RFP signal of OVCAR8 wild type and respective clones grown under 3D and serum free conditions for 48h.

Supplementary Figure 3

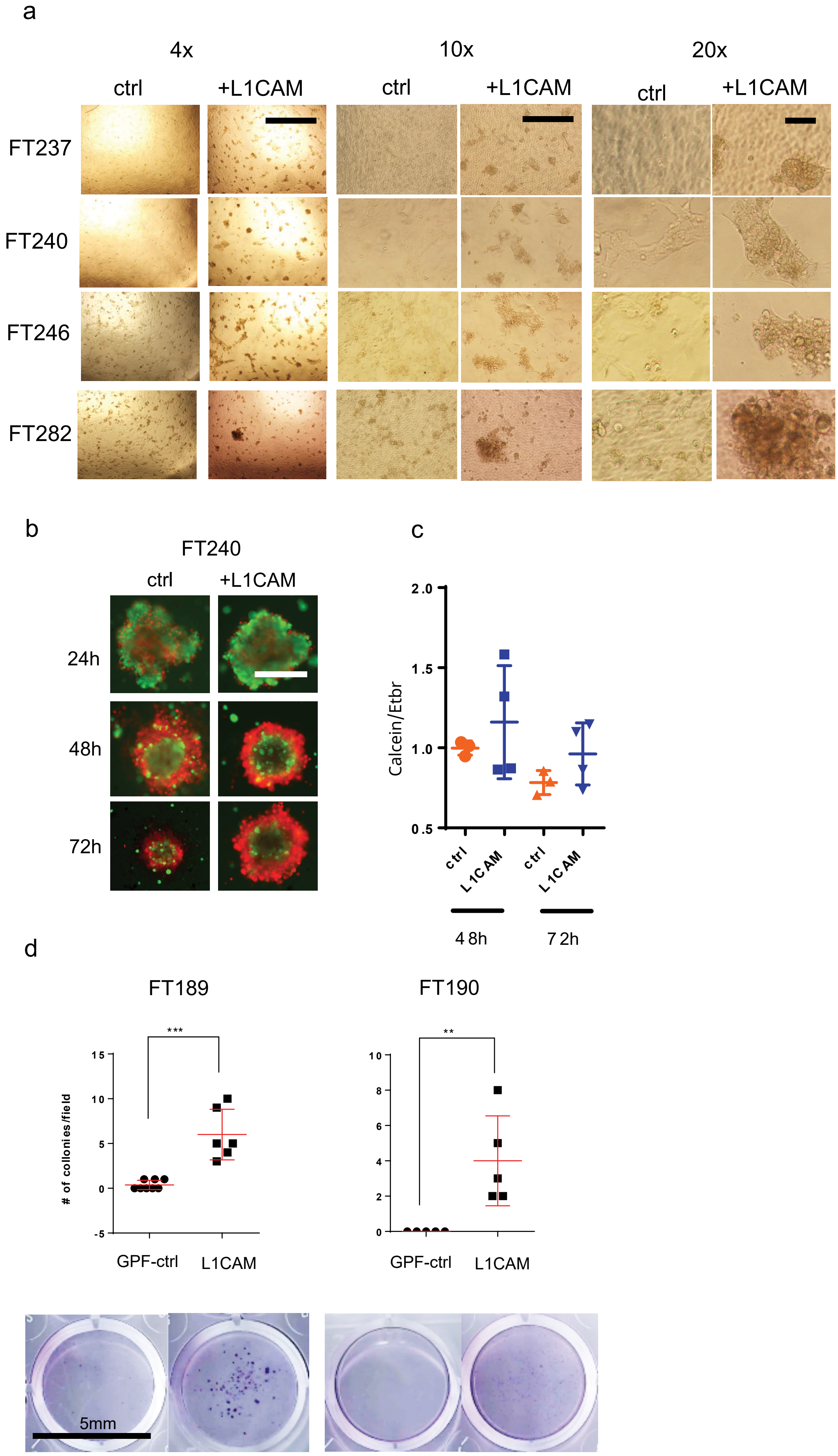

Supplementary Figure 3:

(a) Bright field analysis of FT237, FT240, FT246 and FT282 after transfection with either control or L1CAM vector. 24h after transfection, cells were detached and re-seeded on a new 24 well plate and grown for 48 h before images were taken. (b) Fluorescent images stained with calcein (green) and ethidium bromide (red) representing FT240 cells transfected with a L1CAM (right panel) or a control vector (left panel). Scalebar represents 200 µm. (c) Dot plot depicting the ratio between living and dead cells, assessed by measuring the ratio between calcein (green) and ethidium bromide (red) intensity in FT240 cells transfected with L1CAM or a control vector after culturing under 3D and serum free conditions. (d) FT189 and FT190 were grown under ULA condition for 72 h before seeded to an adherent cell culture plate. Colonies were stained with crystal violet (lower panel) and colonies were counted (upper panel). The experiments were performed trice independently and P-values were calculated with an unpaired two-sided t-test. *=p<0.05, **=p<0.01, ***=p<0.001, ****=p<0.0001

a

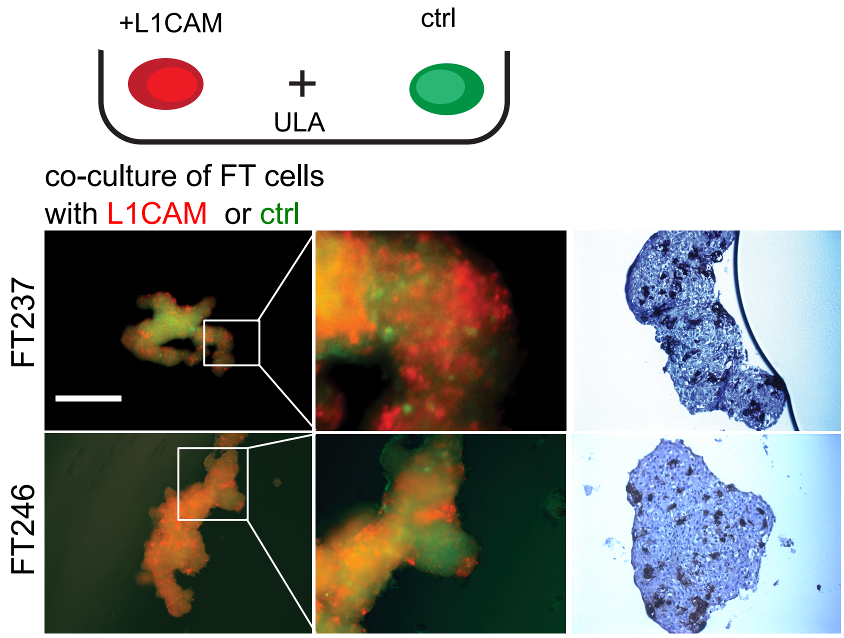

+L1CAM
ctrl
+
ULA
co-culture of FT cells
with L1CAM or ctrl
FT237
FT246

b

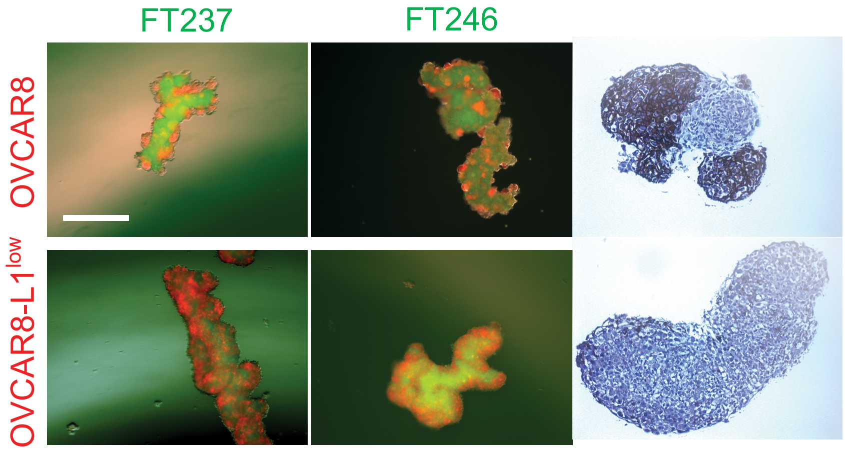

FT237
FT246
OVCAR8
OVCAR8-L1low

Supplementary Figure 4:

(a) Schematic cartoon showing the co-culture experiment under ultra-low adhesion conditions (ULA). Representative fluorescent images of co-culture of FT237 (upper row) or FT246 (lower row) cells that were transfected either with L1CAM (red) or a control vector (green) and co-cultured at a ratio of 1:10, respectively. (b) Co-culture of OVCAR8 (upper row) and OVCAR8-L1^{low} (lower row) that were showing red fluorescent markers with FT237 or FT246 showing fluorescent markers. Scalebar represents 500 µm. All experiments were performed independently three times in triplicate.

Supplementary Figure 5

a

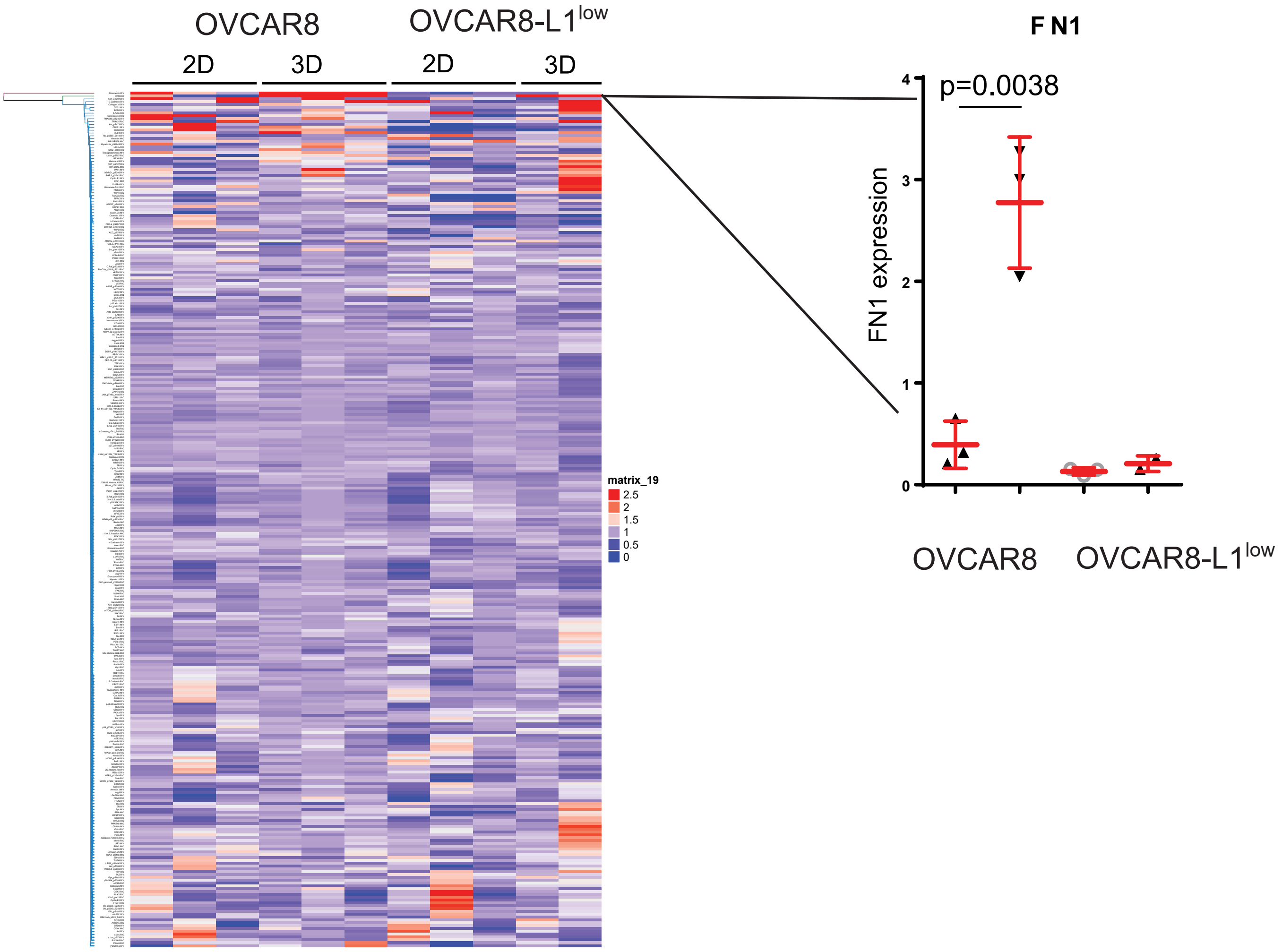

Supplementary Figure 5:

(a) RPPA analysis in OVCAR8 and OVCAR8-L1^{low} under 2D and 3D culturing conditions. (left) centroid clustered heatmap of samples. (right) Dot plot of fibronectin (FN1) expression from RPPA analysis.

Supplementary Figure 6

a

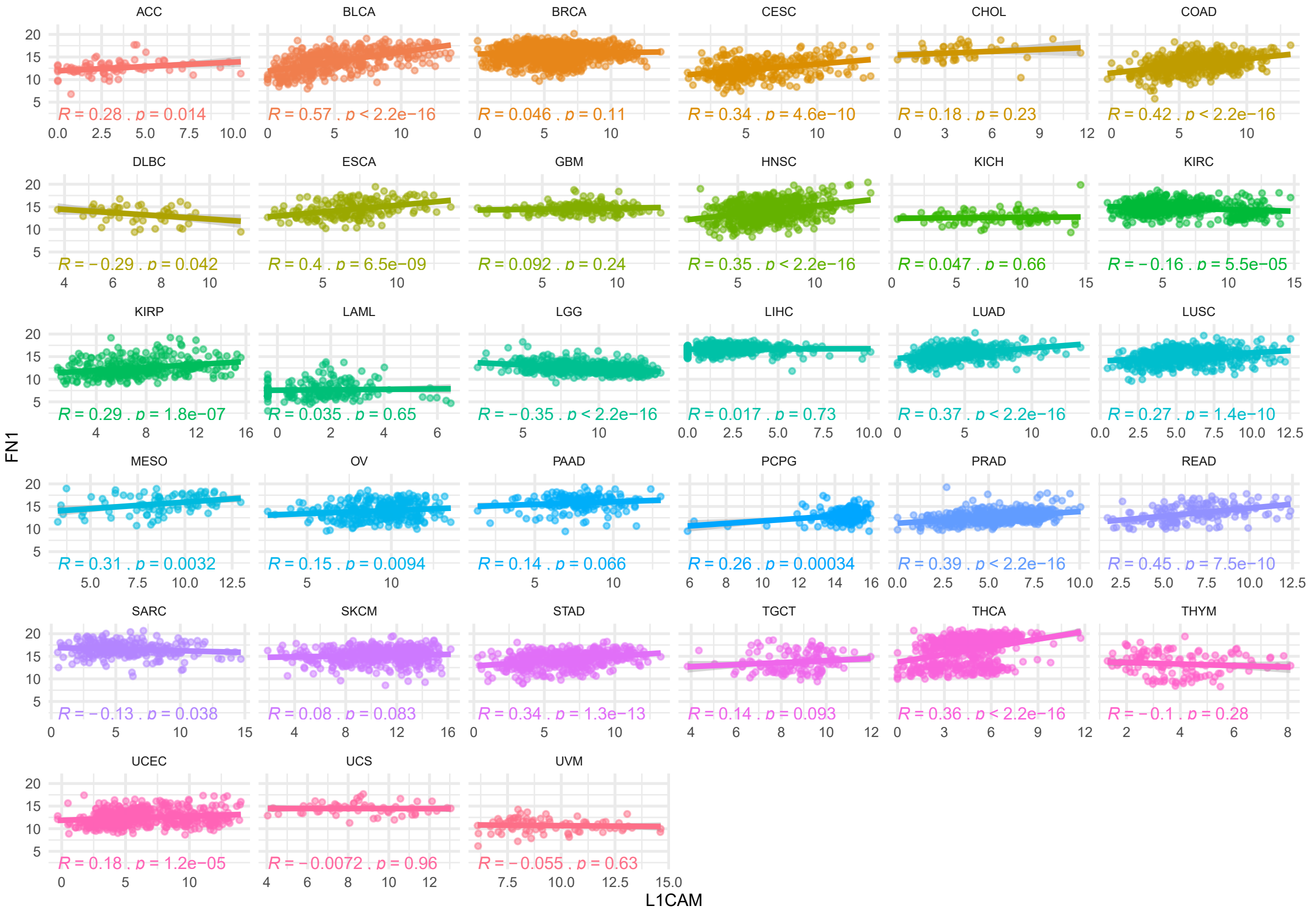

b

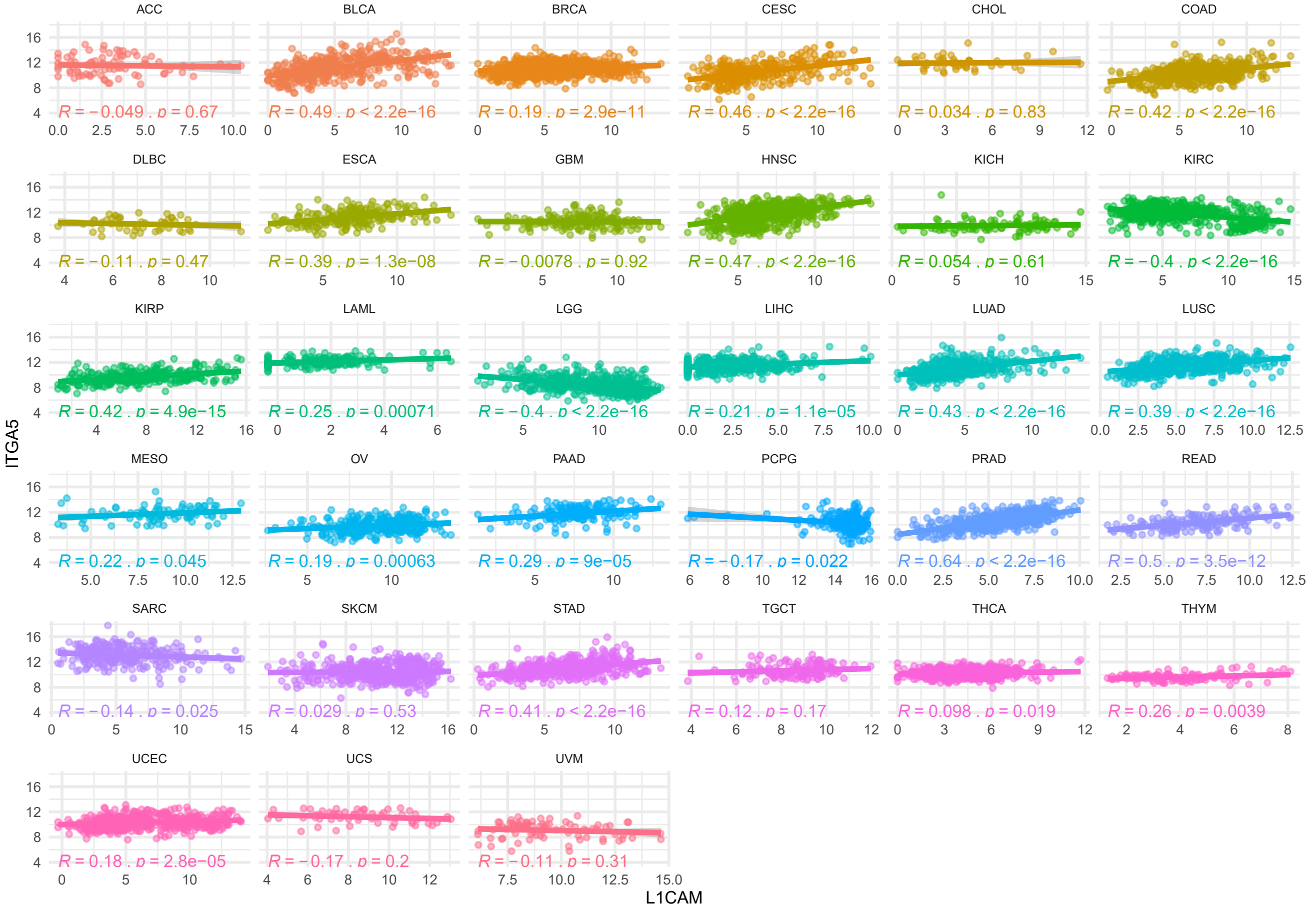

Supplementary Figure 6:

(a and b) Correlation of fibronectin (a, FN1) and Integrin alpha 5 (b, ITGA5) mRNA expression with L1CAM mRNA expression of the Pan-Cancer dataset of the TCGA data. Depicted is Pearson correlation and significance for each cancer type. Cancer type Abbreviations: LAML Acute Myeloid Leukemia, ACC Adrenocortical carcinoma, BLCA Bladder Urothelial Carcinoma, LGG Brain Lower Grade Glioma, BRCA Breast invasive carcinoma, CESC Cervical squamous cell carcinoma and endocervical adenocarcinoma, CHOL Cholangiocarcinoma, COAD Colon adenocarcinoma, ESCA Esophageal carcinoma, GBM Glioblastoma multiforme, HNSC Head and Neck squamous cell carcinoma, KICH Kidney Chromophobe, KIRC Kidney renal clear cell carcinoma, KIRP Kidney renal papillary cell carcinoma, LIHC Liver hepatocellular carcinoma, LUAD Lung adenocarcinoma, LUSC Lung squamous cell carcinoma, DLBC Lymphoid Neoplasm Diffuse Large B-cell Lymphoma, MESO Mesothelioma, OV Ovarian serous cystadenocarcinoma, PAAD Pancreatic adenocarcinoma, PCPG Pheochromocytoma and Paraganglioma, PRAD Prostate adenocarcinoma, READ Rectum adenocarcinoma, SARC Sarcoma, SKCM Skin Cutaneous Melanoma, STAD Stomach adenocarcinoma, TGCT Testicular Germ Cell Tumors, THYM Thymoma, THCA Thyroid carcinoma, UCS Uterine Carcinosarcoma, UCEC Uterine Corpus Endometrial Carcinoma, UVM Uveal Melanoma.

Supplementary Figure 7

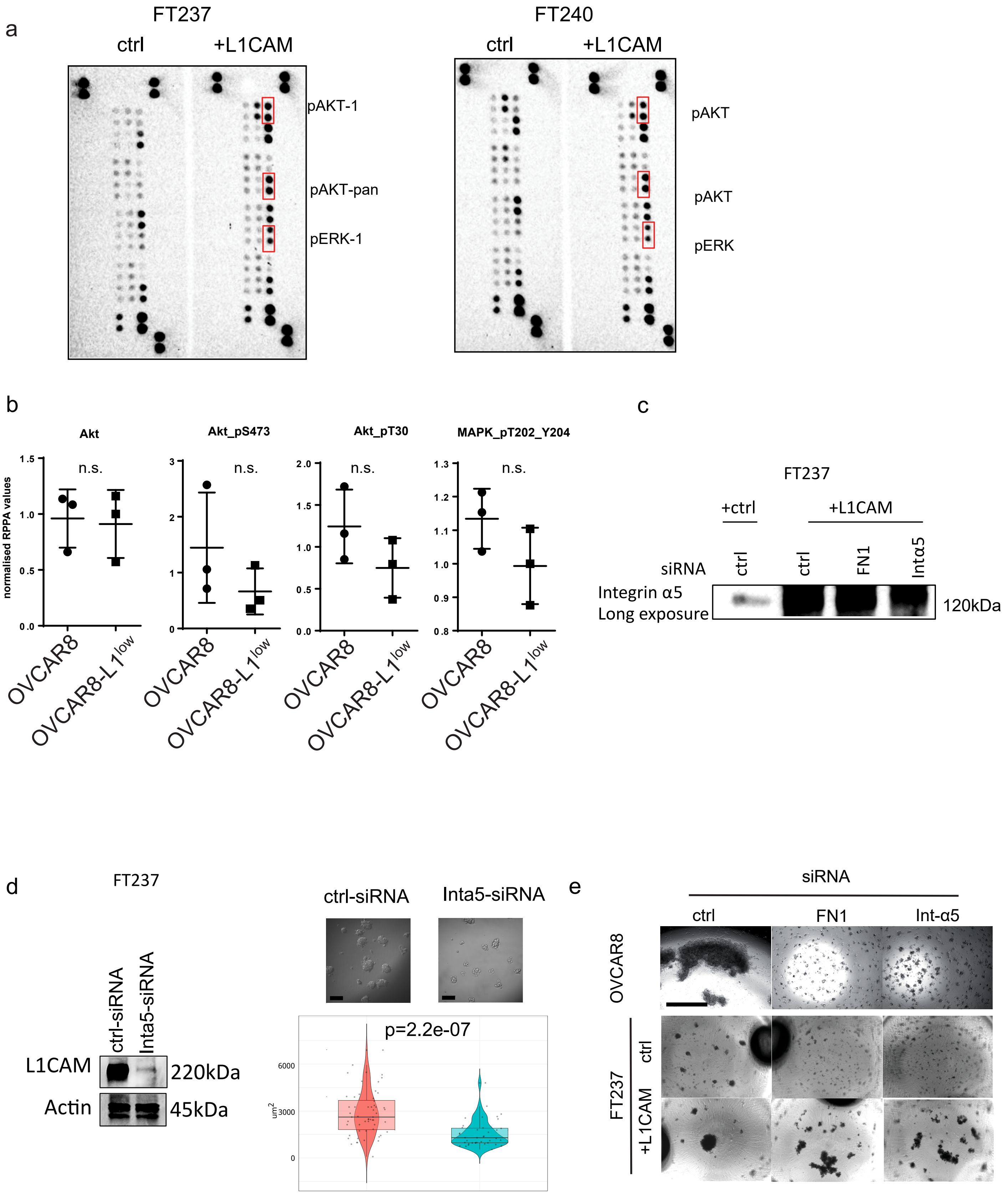

Supplementary Figure 7:

(a) MAPK arrays incubated with lysates of FT237 (left) or FT240 (right) transfected with a control or L1CAM vector. (b) RPPA analysis of AKT, AKT_pS473, AKT_pT30, and MAPK_pT02_Y204 in OVCAR8 and OVCAR8-L1^{low} cultured under 2D conditions. (c) Overexposed Western blot analysis with an antibody against integrin alpha 5 that corresponds to Figure 7G. (d) Left: Western blot analysis with antibodies against integrin alpha 5 and beta-Actin of FT237 transfected with control siRNA or siRNA against Integrin alpha 5. Upper: representative bright field image of cells grown under ULA conditions in serum free media. Below: quantified sphere size. Scalebar represents 500 µm. (e) Bright field images representing OVCAR8, FT237-ctrl and FT237+L1CAM cells grown under ULA conditions in serum free media, 72h after siRNA -mediated attenuation of fibronectin or Integrin α5. Scalebar represents 1000 µm.

Supplementary Figure 8:
Uncropped versions of blots

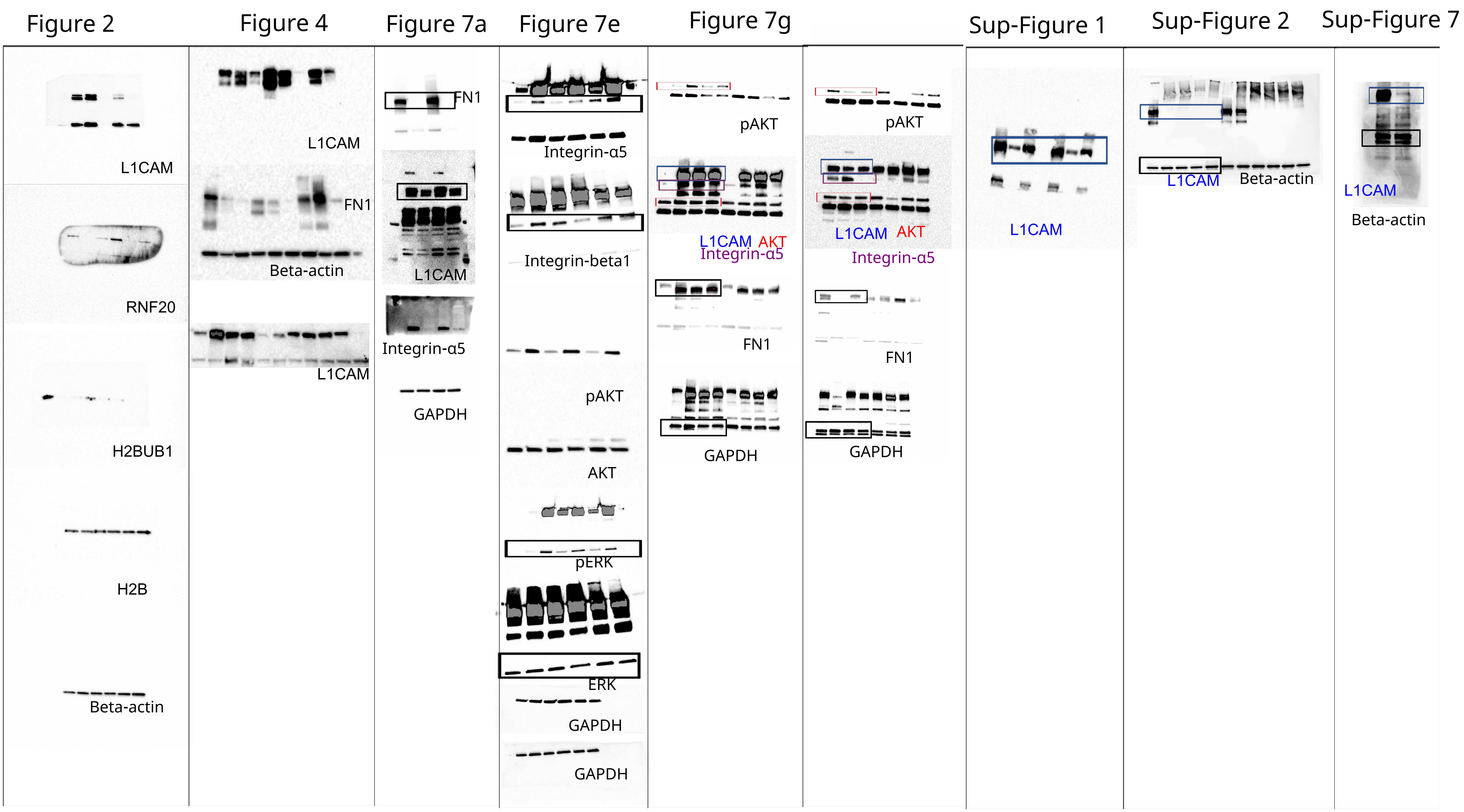

Supplementary Figure 8:

Uncropped blots
