# Supplementary material for: L1CAM is required for early dissemination of fallopian tube carcinoma precursors to the ovary
Source: Commun Biol. 2022 Dec 12;5:1362. doi: 10.1038/s42003-022-04314-8 (PMC9744873; doi:10.1038/s42003-022-04314-8)
Supplement: Supplementary file 3 — Description of Additional Supplementary Files [file 42003_2022_4314_MOESM3_ESM.pdf]

Description of Additional Supplementary Files

File name: Supplementary Data 1

Description: RNAseq analysis of enriched genes upon knockdown of RNF20 and H2Bub1 in fallopian tube cells.

File name: Supplementary Data 2

Description: Analysis of enriched pathways upon knockdown of RNF20 and H2Bub1 in fallopian tube cells.

File name: Supplementary Data 3

Description: Cell line names, origins, and media used in the study.

File name: Supplementary Data 4

Description: Chemicals, plasmids, and recombinant proteins used in the study.

File name: Supplementary Data 5

Description: Antibodies used in the study with manufacturer name, lot number, species, and dilutions.

File name: Supplementary Data 6

Description: All source data underlying the graphs and charts presented in the main figures.
