# Supplementary material for: L1CAM is required for early dissemination of fallopian tube carcinoma precursors to the ovary
Source: Commun Biol. 2022 Dec 12;5:1362. doi: 10.1038/s42003-022-04314-8 (PMC9744873; doi:10.1038/s42003-022-04314-8)
Supplement: Supplementary file 6 — Reporting Summary [file 42003_2022_4314_MOESM6_ESM.pdf]

nature portfolio

Corresponding author(s): Ronny Drapkin

Last updated by author(s): Sep 28, 2022

Reporting Summary

Nature Portfolio wishes to improve the reproducibility of the work that we publish. This form provides structure for consistency and transparency in reporting. For further information on Nature Portfolio policies, see our Editorial Policies and the Editorial Policy Checklist.

Statistics

For all statistical analyses, confirm that the following items are present in the figure legend, table legend, main text, or Methods section.

n/a	Confirmed	
☐	☒	The exact sample size (*n*) for each experimental group/condition, given as a discrete number and unit of measurement
☐	☒	A statement on whether measurements were taken from distinct samples or whether the same sample was measured repeatedly
☐	☒	The statistical test(s) used AND whether they are one- or two-sided<br>*Only common tests should be described solely by name; describe more complex techniques in the Methods section.*
☒	☐	A description of all covariates tested
☐	☒	A description of any assumptions or corrections, such as tests of normality and adjustment for multiple comparisons
☐	☒	A full description of the statistical parameters including central tendency (e.g. means) or other basic estimates (e.g. regression coefficient) AND variation (e.g. standard deviation) or associated estimates of uncertainty (e.g. confidence intervals)
☐	☒	For null hypothesis testing, the test statistic (e.g. *F*, *t*, *r*) with confidence intervals, effect sizes, degrees of freedom and *P* value noted<br>*Give P values as exact values whenever suitable.*
☒	☐	For Bayesian analysis, information on the choice of priors and Markov chain Monte Carlo settings
☒	☐	For hierarchical and complex designs, identification of the appropriate level for tests and full reporting of outcomes
☐	☒	Estimates of effect sizes (e.g. Cohen's *d*, Pearson's *r*), indicating how they were calculated

Our web collection on statistics for biologists contains articles on many of the points above.

Software and code

Policy information about availability of computer code

Data collection	Data from the TCGA database were extracted and downloaded from the XENA portal of the University of California, Santa Cruz (http://xena.ucsc.edu/). The extracted copy number and RNAseq data from the TCGA ovarian cancer cohort, TCGA pan-cancer cohort and the Cancer Cell Line Encyclopedia were analyzed with the GraphPad Prism or the ggplot2 package of the R software.
Data analysis	Data were analyzed with the GraphPad Prism or the ggplot2 package of the R software.

For manuscripts utilizing custom algorithms or software that are central to the research but not yet described in published literature, software must be made available to editors and reviewers. We strongly encourage code deposition in a community repository (e.g. GitHub). See the Nature Portfolio guidelines for submitting code & software for further information.

Data

Policy information about availability of data

All manuscripts must include a data availability statement. This statement should provide the following information, where applicable:

- Accession codes, unique identifiers, or web links for publicly available datasets
- A description of any restrictions on data availability
- For clinical datasets or third party data, please ensure that the statement adheres to our policy

The RNA-seq data presented in this study were submitted to the Gene-Expression Omnibus and can be accessed by the accession number GSE122238 as described in Hooda et al25.

The RPPA data are available through the figshare website and the link https://doi.org/10.6084/m9.figshare.12264404.v1.

Human research participants

Policy information about studies involving human research participants and Sex and Gender in Research.

Reporting on sex and gender	n/a
Population characteristics	n/a
Recruitment	n/a
Ethics oversight	n/a

Note that full information on the approval of the study protocol must also be provided in the manuscript.

Field-specific reporting

Please select the one below that is the best fit for your research. If you are not sure, read the appropriate sections before making your selection.

☒ Life sciences ☐ Behavioural & social sciences ☐ Ecological, evolutionary & environmental sciences

For a reference copy of the document with all sections, see nature.com/documents/nr-reporting-summary-flat.pdf

Life sciences study design

All studies must disclose on these points even when the disclosure is negative.

Sample size	No statistical method was used to predetermine sample size.
Data exclusions	no data was excluded.
Replication	Replication Reported results were tested and confirmed in at least two independent experiments (i.e. minimum two biological replicates).
Randomization	No human participants or animal models were reported in this manuscript.<br>Samples were organized into different treatments conditions.
Blinding	Investigators were blinded when analyzing ovary invasion.

Reporting for specific materials, systems and methods

We require information from authors about some types of materials, experimental systems and methods used in many studies. Here, indicate whether each material, system or method listed is relevant to your study. If you are not sure if a list item applies to your research, read the appropriate section before selecting a response.

Materials & experimental systems

n/a	Involved in the study	
☐	☒	Antibodies
☐	☒	Eukaryotic cell lines
☒	☐	Palaeontology and archaeology
☒	☐	Animals and other organisms
☐	☒	Clinical data
☒	☐	Dual use research of concern

Methods

n/a	Involved in the study	
☒	☐	ChIP-seq
☐	☒	Flow cytometry
☒	☐	MRI-based neuroimaging

Antibodies

Antibodies used	L1CAM 14.10 Peter Altevogt DKFZ<br>L1CAM 9.3 Peter Altevogt DKFZ<br>L1CAM Abcam ab208155<br>β-Actin Cell Signaling #3700<br>Integrin α5 Cell Signaling #98204

Integrin β1 Cell Signaling #34971
Fibronectin 1 Abcam ab2413
AKT   Cell Signaling #2920
p-AKT   Cell Signaling #4060
ERK   Cell Signaling #4695
p-ERK   Cell Signaling #4370
P53   Santa Cruz Biotechnology sc-126
PAX8   Cell Signaling #8875
Stathmin Cell Signaling #3352
P16   Ventana Laboratories 725-4713
control IgG Cell Signaling #5415
control IgG Cell Signaling #3900
GAPDH Cell Signaling #5014

Validation

L1CAM 9.3 Monoclonal antibody validated for IF, IHC FACS in 6 publications. We cited this antibody in Int J Cancer. 2015 Mar 1;136(5):E326-39.
L1CAM 14.10 Monoclonal antibody validated for IF, IHC FACS . First described in Huszar et al. Hum Pathol. 2006 Aug;37(8):1000-8. Epub 2006 Jun 21. In use diagnostically Br J Cancer. 2016 Sep 6; 115(6): 716–724. We cited this antibody in Int J Cancer. 2015 Mar 1;136(5):E326-39.
L1CAM   Validated by the company and cited in 5 research papers
β-Actin Validated by the company and cited in 2919 research papers
Integrin α5 Validated by the company and cited in 12 research papers
Integrin β1 Validated by the company and cited in 35 research papers
Fibronectin 1 Validated by the company and cited in 643 research papers
AKT   Validated by the company and cited in 1132 research papers
p-AKT   Validated by the company and cited in 7806 research papers
ERK   Validated by the company and cited in 4761 research papers
p-ERK   Validated by the company and cited in 6310 research papers
P53   Validated by the company and cited in 79 research papers
PAX8   Validated by the company and cited in 7 research papers
Stathmin Validated by the company and cited in 52 research papers
P16   Validated by the company for diagnostic use.
control IgG Validated by the company and cited in 260 research papers
control IgG Validated by the company and cited in 459 research papers
GAPDH Validated by the company and cited in 32 research papers

Eukaryotic cell lines

Policy information about cell lines and Sex and Gender in Research

Cell line source(s)

Fallopian tube cell lines Drapkin lab
DF-cell lines   Drapkin lab
TOV21G   ATCC
OVCAR4   NCI/DCTD
OVCAR8   NCI/DCTD
COV318   ATCC
OVSAHO   Gottfried Konecny
CAOV3   ATCC
Kuramochi   Gottfied Konecny
CAOV4   ATCC
JHOS-2   RIKEN
JHOS-4   RIKEN
OVKATE   HSRRB

Authentication

Purchased cell lines were authenticated by STR method

Mycoplasma contamination

All cell lines used in this study were negative for mycoplasma. Cell lines were regularly checked by PCR for the presence of mycoplasma

Commonly misidentified lines
(See ICLAC register)

No commonly misidentified cell lines were used in this study.

Clinical data

Policy information about clinical studies
All manuscripts should comply with the ICMJE guidelines for publication of clinical research and a completed CONSORT checklist must be included with all submissions.

Clinical trial registration

n/a

Study protocol

n/a

Data collection

n/a

Outcomes	n/a

Flow Cytometry

Plots

Confirm that:

☒ The axis labels state the marker and fluorochrome used (e.g. CD4-FITC).

☒ The axis scales are clearly visible. Include numbers along axes only for bottom left plot of group (a 'group' is an analysis of identical markers).

☒ All plots are contour plots with outliers or pseudocolor plots.

☒ A numerical value for number of cells or percentage (with statistics) is provided.

Methodology

Sample preparation	Trypsinized cells were washed with FACS buffer (PBS containing 2% BSA) and incubated for 1 hrs with mAb L1 -11A against L1CAM or a IgG isotype control antibody. After two times washing, the cells were incubated with a secondary anti mouse antibody that was conjugated with Alexa488. After three washing steps, the stained cells were analyzed with a FACS Canto II, using Flowing software (Cell Imaging and Cytometry core and Biocenter, Finland) and the R software. Postive cells were calculated relative to the analyzed isotype control.
Instrument	FACS Canto II
Software	Flowing software (Cell Imaging and Cytometry core and Biocenter, Finland) and the R software
Cell population abundance	Full cell populations were analyzed
Gating strategy	All flow cytometry experiments were gated and analyzed similarly.<br>Same gates were used for the control and experimental conditions.<br>Only one gate for FSC and SSC was used to identify main population.

☒ Tick this box to confirm that a figure exemplifying the gating strategy is provided in the Supplementary Information.
